# Specific Association of Teratogen and Toxicant Metals in Hair of Newborns with Congenital Birth Defects or Developmentally Premature Birth in a Cohort of Couples with Documented Parental Exposure to Military Attacks: Observational Study at Al Shifa Hospital, Gaza, Palestine

**DOI:** 10.3390/ijerph110505208

**Published:** 2014-05-14

**Authors:** Paola Manduca, Awny Naim, Simona Signoriello

**Affiliations:** 1 Dept. Scienze della Terra, Ambientali e della Vita, University of Genoa, Genova 16132, Italy; 2 Palestinina Energy and Natural Resources Authority, Gaza City, Palestine; E-Mail: awnynaim@gmail.com; 3 Department of Medical Health and Preventive Medicine, Second University of Naples, Napoli 80100, Italy; E-Mail: simona.signoriello@unina2.it

**Keywords:** major structural birth defects, prematurity at birth, *in utero* metal contamination, newborn hair analysis by DRC-ICP-MS, exposure of mothers to weaponry, correlation of phenotypes with maternal exposure and metal hair load

## Abstract

This study was undertaken in Gaza, Palestine, in a cohort of babies born in 2011. Hair samples of newborns were analyzed for metal load by DRC-ICP-MS. We report specific level of contamination by teratogen/toxicants metals of newborn babies, environmentally unexposed, according to their phenotypes at birth: normal full term babies, birth defects or developmentally premature. The occurrence of birth defects was previously shown to be correlated in this cohort to documented exposure of parents to weapons containing metal contaminants, during attacks in 2009. We detect, in significantly higher amounts than in normal babies, different specific teratogen or toxicant elements, known weapons’ components, characteristic for each of birth defect or premature babies. This is the first attempt to our knowledge to directly link a phenotype at birth with the in utero presence of specific teratogen and/or toxicant metals in a cohort with known episodes of acute exposure of parents to environmental contamination by these same metals, in this case delivered by weaponry The babies were conceived 20–25 months after the major known parental exposure; the specific link of newborn phenotypes to war-remnant metal contaminants, suggests that mothers’ contamination persists in time, and that the exposure may have a long term effect.

## 1. Introduction

Exposure to metals can induce transient and stable pathologies, cancer, and affect key developmental steps via epigenetic mechanisms [1,2]. Epigenetics refers to changes of gene expression that do not derive from alterations of the nucleotide sequence of DNA. Epigenetic changes [3,4,5,6,7,8,9] are a landmark during embryonic development and germ cells determination. Interference by many metals with the basic known epigenetic mechanisms, active at these stages, was documented *in vivo* and *in vitro* in environmental and experimental contexts [9,10,11,12,13,14,15,16,17,18,19,20,21]. Much still remains to be learned about the mechanism(s) of action of each metal and of their associations, and about the mechanism involved in the maintenance of their effects along the somatic lineages and about the inheritability of their effects between generations.

The capability to induce phenotypes at birth has been documented for metals in experimental setups in various species: *Drosophila*, avians and mammals. Exposure to industrially produced metals or accidental exposure was shown to induce birth defects and pathologies in human studies [22,23,24] although there is still controversy among epidemiologists about the effects of metals on the incidence or prevalence of birth defects and prematurity (P), mostly due to the difficulty in defining environmental effectors for human populations, the appropriate population and data accuracy in retrospective studies of birth defects because of differences in registration and exclusion criteria [22].

Most metals persist in time in the environment and many of them accumulate in the bodies, suggesting that dispersion of metal powders in the environment may have both immediate and long term effects on reproductive health and cancers.

Concerns from professionals in Afghanistan, Iraq, Lebanon and Gaza have pointed to increases in birth defects and cancers in these areas during the years following wars. These could be long term effects of the assumption of teratogens and mutagens delivered during military attacks and (or) of persistence of war remnants in the environment [25,26]. The consequence of the explosion of ammunitions with heavy metals was originally raised about DU ammunition; in more recent times “metal augmented “ ammunitions, containing powders of various metals, were developed, and used in the wars and attacks in this century [27]. Knowledge of the body burden of metal elements brought into the environment by this weaponry may help defining the nexus, if there is one, between the war-derived contamination and reported damages, imputable to it, to the health of the populations.

In the Gaza situation, various pieces of information useful to investigate this link were published. By proving the fact of the presence in tissues at the site of different types of wounds of a specific metal signature different for each kind of physical damage caused by weaponry it was shown that teratogens and fetotoxicant metal contaminants (Pb, U, Al, Ti, Cu, Sr, Ba, Co, Hg, V, Cs and Sn) were delivered by weapons in the attacks in the summer of 2006 and during Operation Cast Lead in the winter of 2009 [27]. Teratogens were also detected in examined large bomb craters from 2006 and 2009 bombings [28]; among them, barium, a component of weaponry with electro-thermal ignition pulsed power supply (ETIPPS) was present [29]. The phosphorus (WP) ammunitions utilized during Cast Lead in Gaza were found to contain teratogenic metals, including mercury [30]. Ammunitions and bomb explosions, and explosion of the WP shells in the air, cause the spread of their metal load in the environment, where they may persist for a long time. In another post-war context, other authors [31] and ourselves [32] have shown persisting hair contamination by metals, in adult and children hair, long after major war attacks. In Gaza, ten months after Operation Cast Lead the metal load measured in hair grown in the previous 3–4 months for 57 children showed the presence of toxicant and teratogenic weapon components [33]. Reconstructing the reproductive history of women delivering healthy children in 2011 at Al Shifa Hospital in Gaza, we reported that the prevalence of birth defects has increased in Gaza since 2005, following introduction of air-delivered weaponry [34]. In a recent study [35], we reported highly significant correlations between exposure of parental couples to metal delivering weaponry during Operation Cast Lead, the major recent military attack up to then, and the delivery of a baby with major structural birth defects as defined according to the CD10 [36]. 27% (15/55) of the couples with birth defect children born between May and September of 2011, declared exposure to WP explosions, *versus* 1.7% (67/3,932) of parents with normal offspring born in the same period. Couples with malformed children were also exposed in 18.1% of cases (10/55) to both to WP and bombing and in 20.0% (11/55) of cases to bombing only [35]. Couples with normal children were not asked specifically if they were exposed to bombing. The recall of exposure by the parents of malformed babies was controlled on the OCHA maps and on the data base of the UN Mine Action Team, and resulted accurate. The progeny with birth defects was conceived 22–26 months after exposures. This information is consistent with weaponry being a source of contamination by teratogenic and toxicant metals for the population, and suggest association between reproductive damage, and exposure to attacks. The study described above, did not investigate the metal load of mothers and children with birth defects and could not document a link between specific contamination and end effect and nor inform if phenotypes at birth might derive from genetic or epigenetic changes inducible by environmental teratogens.

In the investigation here reported we attempt to answer some of the questions raised by the correlation observed before between exposure to weaponry and birth defects and directly assess the possible impact of metals in affecting epigenetic changes during embryonic and fetal life. We investigated if there is a higher metal load in babies born with birth defects or prematurely than in normal babies due to metal contamination *in utero*. We determined which are the contaminants present during the embryonic and fetal life of the affected babies and if these are different according with the phenotype. We discuss what our knowledge of the properties of the contaminants found specifically associated to birth defects and prematurity imply about the mechanism, epigenetic or genetic, by which they affect changes of normal development *in utero*. We also show that there is no significant difference of metal load in birth defect children whose parents had documented exposure to attacks and those which were not acutely exposed.

We studied the same cohort where association between exposure of parents to attacks and birth defect was established before, and for which residential history, objectively documented exposure history, reproductive history, pedigrees and final phenotype of children was already reported; here we analyzed the hair of the babies with birth defects from this group, babies born prematurely and normal babies born in the same time span and from which we had collected samples at birth.

By measuring directly the metal load in newborn hair, we focus on contaminants which may have direct effects on embryonic development. The metal load of newborn hair reflects the total accumulation in the period of hair growth in the womb (from about 22 weeks of gestation), excluding direct contamination by the external environment. High metal load in the newborns reveals persisting contamination of the mothers, who act as “metal donors” during gestation. Thus, our approach reduces confounding factors from the external environment and identifies specific elements successfully entered in the body of the embryo-fetus and that may have an impact directly on their growth and morphogenesis *in utero*.

We show here a characteristic pattern of differential accumulation of metals derived from *in utero* exposure, in excess compared to normal babies, for each of birth defect or premature babies. Our data imply that the metals identified in the newborns are persistent contaminants of the population, still capable of affecting the reproductive life at a distance of almost two years from their ascertained delivery by military action.

## 2. Experimental Section

Method of collection of clinical and demographic data, family, reproductive, exposure to war and residential history of the newborns was reported in a previous publication [35]. Permission for the study was obtained from the Ministry of Health in Gaza and from the Ethics Committee at Genoa University (Italy). Hair were collected within few minutes of birth and without any previous treatment besides drying the child’s head with a clean, dry cotton cloth. The hair was taken from the nape of neck, when possible, or on the occipital side and sealed in a plastic bag till analysis by DRC-ICP-MS. Individual exposure during the fetal growth can be thus measured. The cases of newborns with birth defects were 87.3% (48/55) of those born in succession at Al Shifa hospital in Gaza from May to October 2011, all that had enough hair for testing. Normal children (N; at term and equal, or above 2.5 kg at birth) were randomly chosen among 3,892 births and prematures (born before 37 weeks gestational age and less than 2.0 kg in weight) as those with enough hair for testing, among 77 registered in the same 5 months period of study. Blinded analyses were performed, with a standard protocol, at the Laboratories of the Maugeri Foundation (Pavia, Italy), and included additional seven samples of hair with already known metal load, as technical controls. Briefly, hair samples were weighed and mineralized in a microwave oven in 65% HNO_3_ (3.5 mL) + 30% H_2_O_2_ (0.5 mL, Suprapur-Merck), diluted 1:20 in bidistilled H_2_O and analyzed in DRC-ICP-MS with 20 sweeps/reading. The DRC-ICP-MS instrument was an ELAN DRC ІІ (Perkin Elmer, SCIEX Instruments, Toronto, ON, Canada) with a quadrupolar analyzer, mass interval 5 -270 amu, source ICP, radiofrequency 40 MHz, max. power 1,600 W. Elements analyzed were ^7^Li, ^9^Be, ^48^Ti, ^51^V, ^52^Cr,^ 55^Mn, ^59^Co, ^60^Ni, ^63^Cu, ^64^Zn, ^75^As, ^78^Se, ^85^Rb, ^114^Cd, ^120^Sn, ^121^Sb, ^138^Ba, ^140^Ce, ^184^W, ^202^Hg, ^205^Tl, ^208^Pb, ^238^U. Limits of detection (LOD) varied from 0.01 for ^78^Se, to 0.0004 for ^238^U. Statistic analysis: Difference in metal concentrations between groups was assessed by the Wilcoxon-Mann-Whitney test. All analyses were performed with the R software, version 2.15.3 (The R Foundation for Statistical Computing, 2013). Metal concentrations, in ppm, are reported as median values and inter-quartiles ranges for each group.

## 3. Results and Discussion

**Figure 1:**
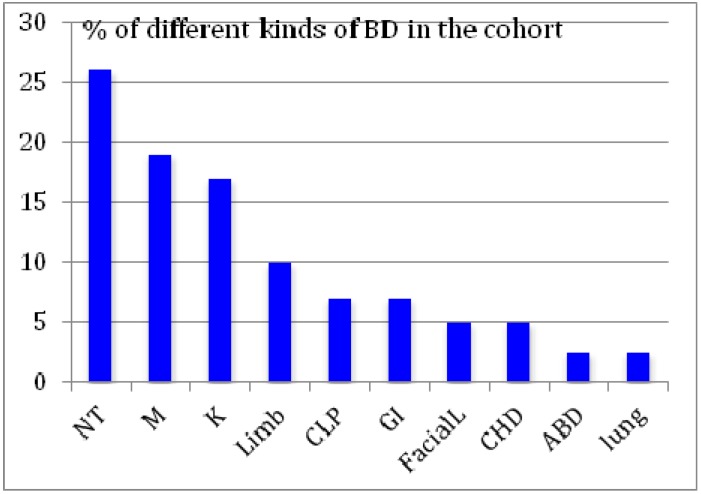
The kind of birth defects presented by the 48 newborns with birth defects whose hair was analyzed for metal load. NT-nuclear tube, M-multiple, K-renal, CLP-cleft lift/palate, GI-gastrointestinal, CHD-congenital heart disease, ABD-abdominal.

The hair of 48 babies with birth defect (BD), and born with hair in the study period, were analyzed for metal load. The cohort included nine cases with a previous BD in the family, of which five were likely to be familiar congenital defects, as inferred from the parent’s and near kin’s reproductive history. Distribution of the incidence of each type of birth defects in the cohort is reported in Figure 1.

Hair was also analyzed from nine babies, among the about 77 preterm babies without birth defects (P), born in a healthy family, and 12 full term healthy babies (N) randomly chosen among the normal babies born in the same time span and without cases of birth defects in the siblings and in the parents’ collaterals.

The residence of all parental couples of the three groups was stable since 2008; in no case was parental exposure to environmental accidents other that war attacks during Operation Cast Lead, or work exposures or smoking of the mother reported. The father of one BD baby was wounded during Cast Lead. Parents of preterm and normal babies studied here declared no direct exposures to WP attacks; were not asked about exposure to bombing. Direct exposure among the 48 couples with BD baby was 50% (24/48) for any attacks by bombs, ammunitions, WP shelling, and/or for rubble collection and returning to damaged housing immediately after the events. Thirteen couples (27.0%) declared no direct involvement in attacks on their house or next door houses and we refer to them as not acutely exposed. Eleven couples did not respond to the section about exposures (22.9%). For the couples “exposed” to attacks there was direct confirmation of their recall by matching residences with the database of the UN mine action team.

Analysis of metal load by DRC-ICP/MS were run for Li, Be, Co, Zn, Hg, Cd, Ba, V, Mn, Sn, Sb, Ti, Ce, Rb, Pb, W, Tl, As, Ni, Se, V, Cr, Cu, U, Mg, K. These metals were chosen on the basis of previous experimental data that showed that toxicant and teratogenic metals on this list were detected as components of weapons, accumulated in people after the attacks of 2009, and because they are therefore considered war remnants; to these was added analysis for selenium, a teratogen when in higher than physiological amounts, which was never tested before. Data for all metals were statistically analyzed for the differences in amounts between groups. We show in Table 1, Table 2 and Table 3 the elements with relevant differences in amounts between the phenotype groups, and some metals known toxicants/endocrine interferents/teratogens, for which instead we did not detect significant differences between groups. All elements analyzed but not shown had no differences for the three groups.

**Table 1:** Comparison of metal load (ppm) between newborn with birth defects (N = 48) and normal (N = 12). Difference in metal concentrations betweengroups was assessed by Wilcoxon-Mann-Whitney test. Interquartile range in brackets.

Metal	Newborn wth BD	Normal newborn	p-value (Wilcoxon-Mann-Whitney)
	Gaza 2011 (N = 48)	Gaza 2011 (N = 12)	
**Sn**	0.23 (0.08–0.54)	0.04 (0.02–0.09)	**0.002**
**Ba**	0.74 (0.51–1.27)	0.60 (0.37–0.73)	0.154
**W**	0.03 (0.02–0.07)	0.02 (0.01–0.04)	0.365
**Hg**	0.93 (0.02–0.95)	0.00 (0.00–0.02)	**0.003**
**Pb**	0.81 (0.49–1.16)	0.60 (0.52–1.21)	0.820
**U**	0.00 (0.00–0.00)	0.00 (0.00–0.00)	0.164
**Se**	0.32 (0.22–0.47)	0.13 (0.09–0.24)	**0.004**
**Sb**	0-03 (0.02–0.06)	0.05 (0.04–0.11)	0.160
**Cd**	0-03 (0.02–0.06)	0.05 (0.03–0.09)	0.143
**Cr**	0.41 (0.29–0.59)	0.78 (0.38–1.17)	0.053

Data are reported as median and interquartile range, Median (IQR).

**Table 2:** Comparison of metal load (ppm) between children with neural tube defects (NT, N=11) and children with polycystic kidney defect (PCK, N=5), the two single body compartment defects most frequent among our patients.

Metal	Newborn NT defect	Newborn PCK defect	*p*-value (Wilcoxon-Mann-Whitney)
	Gaza 2011 (N = 11)	Gaza 2011 (N = 5)	
**Sn**	0.32 (0.14–1.04)	0.15 (0.06–0.30)	0.27
**Ba**	0.64 (0.53–0.70)	0.54 (0.34–0.73)	0.66
**W**	0.03 (0.02–0.08)	0.14 (0.03–0.26)	0.28
**Hg**	0.05 (0.02–0.31)	0.51 (0.17–0.95)	0.16
**Pb**	1.16 (0.79–2.23)	0.74 (0.73–1.75)	0.66
**Se**	0.30 (0.22–0.69)	0.19 (0.16–0.36)	0.39
**Sb**	0.03 (0.02–0.05)	0.04 (0.02–0.08)	0.57
**Cd**	0.03 (0.02–0.07)	0.05 (0.05–0.08)	0.17
**Cr**	0.44 (0.26–0.69)	0.47 (0.343–0.75)	0.38

Note: Data are reported as median and interquartile range, Median (IQR).

BD children, compared to normal children showed significant higher median content of Hg, Sn and Se (Table 1). Comparison between children with neural tube defects (NT, number = 11) and children with polycystic kidney defect (PCK, number = 5), the two most frequent single body compartment defects in our patients showed no significant difference for any of the metals tested (Table 2).

**Figure 2:**
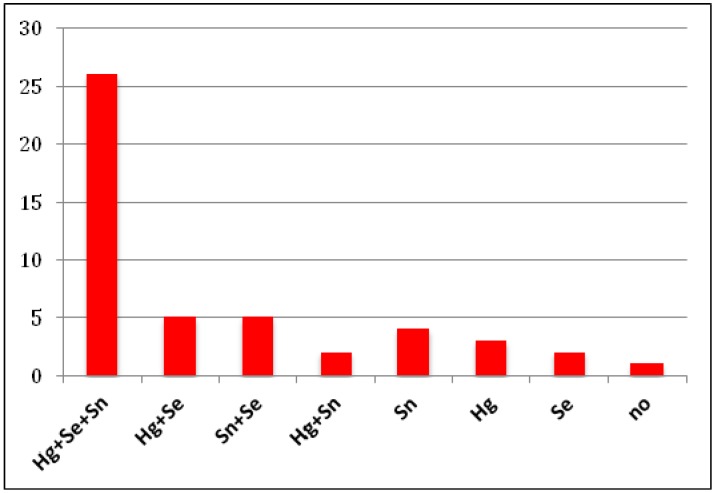
Number of individuals in the cohort of newborns with birth defect with hair load of contaminants higher than the median levels of controls and association of these metals. no = no metal above control.

When considering the individual babies with birth defects, these had higher than median level of normal babies for Hg in 36 cases, for Se in 38 cases and for Sn in 37 cases. These metals were most often detected in association, as shown in Figure 2.

Thus, 26/48 of the babies are contaminated by Hg, Se and Sn together, each in significant high load; individuals with high composite load of Se and Sn (5/48), Hg and Se (5/48) or Hg and Sn (2/48) were also present. Nine children had only one of each of these contaminants; of these, four had only Sn. Only one baby had no significantly higher levels than normal babies for any of these metals.

Comparison of median values from children with BD divided according to ascertained exposure (24 cases) and not acutely exposed (13 cases) showed that they are not significantly different from each other for all the three specific contaminant elements, Hg, Se. Sn, (Table 3, Part A) and that also the non acutely exposed BD show a significant higher content of these compared to normal children (Table 3, Part B).

The definition of not acutely exposed was chosen with reference to the reality of the situation on the ground, whereby even people that were not directly under attack, after the wide destruction following Cast Lead, have routinely walked past demolished housings and in the dirt and rubble around these. Hg can be assumed also through skin, beside than by respiratory and digestive routes.

Comparison of the metal load in hair between preterm born and normal, showed a pattern differing from normal babies, with Sn and Ba in significantly higher amounts, while the amount of Hg did not differ from normal babies, and Se was significantly lower (Table 4).

**Table 3:** **Part A.** Comparison of metal load (ppm) between children with BD whose parents were exposed directly to attacks and their remnants (exposed, N=24) and those without acute exposure (not exposed, N=13). Median values (and Interquartile range) are shown for the relevant contaminant elements. P refers to comparison between the two groups. **Part B.** Comparison of metal load (ppm) between children with BD whose parents were not acutely exposed to attacks (not exposed, N=13) and normal children (N=12). Median values (and Interquartile range) are shown for the relevant contaminant elements. P refers to comparison between the two groups.

A			
Metal	BD exposed (N = 24)	BD not exposed (N = 13)	*p*-value (Wilcoxon-Mann-Witney)
Hg	0.137 (0.029–0.309)	0.087 (0.053–0.206)	0.701
Se	0.273 (0.190–0.558)	0.365 (0.286–0.482)	0.364
Sn	0.262 (0.116–0.591)	0.228 (0.049–0.535)	0.479
**B**			
**Metal**	**BD not exposed (N = 13)**	**Normal (N = 12)**	***p*-value (Wilcoxon-Mann-Witney)**
Hg	0.087 (0.053–0.206)	0.00 (0.00–0.015)	0.005
Se	0.365 (0.286–0.482)	0.132 (0.094–0.238)	0.006
Sn	0.228 (0.049–0.535)	0.042 (0.016–0.093)	0.019

Note: Data are reported as median and interquartile range, Median (IQR).

**Table 4:** Comparison of metal load (ppm) between children prematurely born and normal.

Metal	Normal Newborn	Prematurely Born	
	**Gaza 2011 (N = 12)**	**Gaza 2011 (N = 9)**	***p*-value (Wilcoxon-Mann-Whitney )**
**Sn**	0.04 (0.02–0.09)	0.25 (0.23–0.89)	**0.002**
**Ba**	0.60 (0.37–0.73)	1.07 (0.62–1.58)	**0.03**
**W**	0.02 (0.01–0.03)	0.03 (0.02–0.03)	0.19
**Hg**	0.00 (0.00–0.02)	0.00 (0.00–0.05)	0.47
**Pb**	0.60 (0.52–1.21)	1.06 (0.73–2.10)	0.19
**Se**	0.13 (0.09–0.24)	0.05 (0.00–0.17)	0.16
**Sb**	0.05 < 80.04–0.11)	0.06 (0.02–0.17)	0.55
**Cd**	0.05 (0.03–0.09)	0.08 (0.06–0.09)	0.28
**Cr**	0.78 (0.38–1.17)	0.75 (0.46–0.78)	0.81

Note: Data are reported as median and Interquartile range, *Median (IQR)*.

None of the other teratogens here shown had significant differences of load in hair of the three groups of babies. We are not aware of other studies which directly associate hair metal load in newborns, measuring their effective exposure *in utero*, with their phenotype at birth, in a background where at least 50% of the parental couples with birth defect babies had documented direct exposure to weapons that were shown to deliver teratogens and toxicants.

One of the motivations to conduct this study is that birth defects were shown to increase in Gaza since 2005, and prematurity is also rising, as monitored in the last three years (H. Al Whadia, personal communication).

We thus searched for proof of contamination in the subjects that are the “end point” of the correlation previously reported of “exposure to white phosphorus ammunitions of parents with babies with birth defect, by determining if metals, known to be delivery by weaponry to which parents were exposed, are available *in utero* to their progeny.

By analysis of newborns we eliminated confounding effects from any exposure after birth of the baby, and the uncertainty on interpretations of the results associated to the differential passage of metals through the placenta [37,38,39,40], P. Manduca, unpublished data). In principle this does not rule out that other teratogens and toxicants may also be present *in utero* and do not reach the baby hair or that contaminants that may not reach the uterus could be affecting the mothers’ health. Nonetheless, the specificity of association of a different combination of contaminants in high load *in utero* with either birth defect or prematurity, implies that the contaminants we have detected directly affect the developing embryo-fetus.

The metals found in hair of newborn in excess amounts are on the one hand side candidate effectors of epigenetic developmental changes and candidates for causing long term health effects because of their persistence in the environment and accumulation in organisms, and could produce damages that can be trans-generationally transmitted [41]. On the other hand, these metals were all, with the exception of selenium, which was not tested before, detected in weaponry and documented in wounds after attacks in Gaza, and one year thereafter in the environment and people’s hair.

Mercury is a teratogen, with no known mutagenic action; it transpasses the placenta [37,38,39,40], interferes in key pathways in development [42,43,44]: Notch pathway [42], modulation of key enzymatic antioxidant and oxidative stress makers [44,45]; it affects sperm phenotypes [46], and various organ compartments during rat embryogenesis [47,48], and is neurotoxic [17,49]. Mercury is an endocrine disruptor and affects spermatogenesis, causes malformations and reproductive damages in mammals and human [10,50]. Mercury affects DNA methylation, also via DMT1 down-regulation, affects histones methylation and acetylation [21], cell signaling, and expression of specific genes [51,52,53], having multiple mechanisms for interference in cell growth, survival and differentiation, interference in organ homeostasis and developmental processes. Inherited phenotypic changes consequent to previous Hg exposure were described on neurological phenotypes *in vivo* [54].

Selenium is a metalloid with both toxicological and nutritional properties. Observational and experimental human studies showed that toxicity may occur at much lower levels of Se than previously surmised [55]. At concentrations above physiological, Se is a teratogen, more efficient than Hg itself, and synergic with it, in birds and rats [55,56]. Selenium is also used in cancer therapy for its action on the redox system, and its pro-apototic effect [57]. It affects the function of heat shock proteins, intracellular signaling, is an estrogen modifier, modulates the effects of other metal toxicants; all these functions are compatible with its capability to interfere with multiple pathways during in cell growth and embryo morphogenesis [58,59].

Babies with BD have specifically a higher load than normal babies, and of the level considered safe, of Hg, Se and Sn. Most frequently (26/48) the presence of the three metals together that correlates to BD. Five other individuals had high load of both Hg and Se, and 7 of Hg or Se, with Sn. This suggests synergism in teratogen action. Synergism was reported for Hg and Se in avian development and in embryo toxicity in rat [60]. Single contamination for either Hg or Se was in five individuals, and only five individuals in the cohort had no contamination by either Se or Hg.

The contamination is common to BD babies from couples with documented direct exposure and from not acutely exposed ones. The fact that contamination for Hg was found also in the BD babies whose parents were not acutely exposed to the attacks is an indication of the widespread diffusion of war remnants and of the risk that their assumption poses on terms of reproductive health. Widespread diffusion in the environment is not necessarily surprising for Hg, a component of WP ammunitions, which explode in the air, potentially diffusing their metal content in a wide area and that can be also assumed by the skin. It also suggests that the ammunitions containing Hg may be most effective in determining reproductive damages. The finding of the metal signature also in BD babies of not acutely exposed mothers worries because suggests that continual assumption and accumulation may still be occurring, a point on which presently we have no data, but that should be investigated.

We do not have a specific guess on why different kinds of BD, affecting different compartments of the embryo in development have similar levels and kind of contaminants, as shown by the comparison of the small number of neural tube with kidney malformations. Analysis of a higher number of cases will give more insight on this point and increase the significance of this comparison.

Tin transpasses the placenta and causes inhibition of hemooxygenase in the fetus; it acts as estrogen interferent and at high environmental concentrations is positively associated to congenital neural tube defects [61]. Barium is a toxicant and nephropathy was observed in rodents following long-term oral exposure [62]; its transfer from mother to child is not quantitative (P. Manduca, unpublished data).

Prematurity is on the increase in many countries, often associated to industrial, chemical agriculture and traffic increases, and subject of prevention campaigns, nonetheless we are not aware of studies reporting metal load in premature babies. Here, preterm babies without malformations have a different metal load than normal and BD babies, with high levels of Sn and of Ba and lower than normal load of Se. The relevance of the combination of high load of Sn and Ba, and lower than normal load of Se in preterm babies needs further investigation. The fact that Ba is not passively diffused through the placenta, suggests that mothers of P babies have an even higher load than the child, which could also affect their or the child health and the capability to carry pregnancy to full term.

The data suggest that a high load of Sn is not teratogen *per se*, while it could contribute to fetal suffering and preterm delivery, in agreement with what is known of the effects of Sn. In agreement with this, among the four couples with BD babies with only a high load of Sn, three had a previous congenital BD in the family; thus these BD may be inherited; the only baby with BD and no relevant hair contamination or family history of BD, may occur as sporadic, random new event.

The findings also suggest that the effects on reproductive health are “potentiated” when two or three specific metals are concomitantly present for either phenotype. It should be investigated if the relative amounts of these metals are influenced by modality of reciprocal regulation of levels, as suggested in some experimental setting to occur for Se and Hg [40,52,55,58,60].

Direct exposure *in utero*, specific association to phenotype, together with the know characteristic of action of each group of metals, leads us to interpreter our results in the key of epigenetics effects for birth defects and respectively as toxicant effects on mother, or both mother and child, for premature babies, due to the exposure during embryonic-fetal growth.

In all cases, the donor of contaminants are the mothers, but we cannot say if this is due to continuing exposure from a still contaminated environment or to release during the metabolic changes of pregnancy of contaminants previously accumulated in their bodies.

The exceeding load detected in association to BD and preterm is for specific metals components of weaponry already documented in Gaza after military attacks in the 2006 and 2008/09.

The other major potential source of toxic and teratogen metals in Gaza could be waste mismanagement, a collateral effect itself of war, ensuing bombing and impossibility to reconstruct the waste treatment plants because of the ongoing blockade. Theoretically, this might be the second source of metal dispersal in the environment of Gaza. The association of, only and the same, specific contaminants with the birth defects also in families were there was not documented exposure to attacks, nonetheless, inclines us to think that more than a generalized effect due to metal contaminants in waste, these families may have happened to live near, or spend time in places were the remnants of attacks were present.

Industry is very limited and the use of pesticides/insecticides was excluded as relevant according to the answers provided by the couples, so these last two are less likely sources for any metal contaminant.

The high frequency of direct exposure to attacks in 2008/09 for the parents of BD children and their stable residence afterwards in the areas of attacks, concur to support that the source of contamination in these babies is their mother assumption of war-dispersed teratogen metals, and thus the phenotype of their progeny would be a consequence.

The fact that this consequence was recorded in pregnancies occurring no less than 22–26 months after the acute exposure of the mother, points to the long term effect of the exposure. At this time we cannot distinguish if this could be an effect of accumulation due to acute exposure, or of continual assumption from a still contaminated environment up to 2011, or both. Also effects of metals on the functionality of gametes, could occur under these circumstances, and will be worth investigating, as it can be done in non-invasive ways on sperm.

The parents of the P group had no direct exposure to WP attacks during Cast Lead, although they lived in areas surrounding those bombed. As we did not ask in our previous data collection if they were directly exposed to bombing we cannot trace objectively the likely source of the metals in the history of these families. As for the cases of not acutely exposed BD, we have to consider here that wide areas were potentially contaminated by weaponry-derived metals and that the daily life put people in more or less frequent contact with war remnants in time, according to their daily business and routines.

Contamination of a population is a challenging burden for the countries affected. Identification first, and then in depth research for modalities of action of the major toxicants involved, are needed to plan remediation for the victims before pregnancy, and for care during it, and to provide information for public health.

Here, we have, for the first time, identified potential effectors of birth defect phenotypes; this allows to design *ex vivo* or *in vitro* molecular studies to document changes expected in the DNA methylation, transcriptome and proteome and to investigate about cause-effect relationships and predispose tests for remedies.

## 4. Conclusions

In summary, focusing on the analysis of metal contamination during *in utero* growth we show for the first time specific association of high teratogen/toxicant metal load with a phenotype at birth. By testing environmentally unexposed newborns, we eliminated confounding factors. Our data implicate specific metals and associations of these, in the induction of specific damages during fetal growth. They also suggest that contamination by a combination of specific teratogens or toxicants may better work as inducer of phenotypes. The likelihood that the contaminants derive from exposure to weaponry or to war remnants is high. The measure of contamination in newborn with birth defects or premature two years after the acute exposure of mothers points to long term effect of the environmental changes introduced by attacks in the progeny of directly exposed subjects.

A general consideration that we wish to offer is that in studies following wars (or catastrophes), even when metals or other remnants are not found in soil or short living organisms sometime after the event, lack of their finding does not necessarily reassure about the future health of people, and long term effects could be still expected; therefore the assessment of metal load in these circumstances should be directly on people.

The major limit of this study is that, while offering proof of fact of the association of the specific phenotype, birth defect or prematurity, with contamination by specific metals, known war contaminants, it is not, strictly speaking provding a mechanistic cause–effect demonstration.
